# The analgesic efficacy of intravenous regional anesthesia with a forearm versus conventional upper arm tourniquet: a systematic review

**DOI:** 10.1186/s12871-018-0550-4

**Published:** 2018-07-18

**Authors:** Valerie Dekoninck, Yasmine Hoydonckx, Marc Van de Velde, Jean-Paul Ory, Jasperina Dubois, Luc Jamaer, Hassanin Jalil, Björn Stessel

**Affiliations:** 1 Department of Anesthesiology and Pain Medicine Jessa Hospital, Virga Jesse Campus, Stadsomvaart 11, 3500 Hasselt, Belgium; 2 Department of Cardiovascular Sciences, KU Leuven and Department of Anesthesiology, UZ Leuven, Leuven, Belgium; 3 Department of Anesthesiology, Maastricht University Medical Center, Maastricht, The Netherlands

**Keywords:** Intravenous regional anesthesia, Forearm IVRA, Upper arm IVRA, Analgesic efficacy, Bier block

## Abstract

**Background:**

The main objective of this review is to perform a systematic review and meta-analysis of the existing evidence related to the analgesic efficacy with the use of conventional, upper arm intravenous regional anesthesia (IVRA) as compared to a modified, forearm IVRA in adult patients undergoing procedures on the distal upper extremity.

**Methods:**

MEDLINE, EMBASE and CENTRAL (Cochrane) databases were searched for randomized controlled trials published in English, French, Dutch, German or Spanish language. Primary outcomes of interest including description of quality level of anesthesia and onset of sensory block were assessed for this review. Dosage of the local anesthetic, local anesthetic toxicity and need for sedation due to tourniquet pain were considered as secondary outcomes.

**Results:**

Our literature search yielded 3 papers for qualitative synthesis. Four other articles were added into a parallel analysis of 7 reports that provided data on the incidence of complications and success rate after forearm IVRA. Forearm IVRA was found to be as efficient as upper arm IVRA (RR = 0.98 [0.93, 1.05], *P* = 0.78), but comes with the advantage of a lower need for sedation due to less tourniquet pain.

**Conclusion:**

Our results demonstrate that forearm IVRA is as effective in providing a surgical block as compared to a conventional upper arm IVRA, even with a reduced, non-toxic dosage of local anesthetic. No severe complications were associated with the use of a forearm IVRA. Other benefits of the modified technique include a faster onset of sensory block, better tourniquet tolerance and a dryer surgical field.

**Registration of the systematic review:**

A review protocol was published in the PROSPERO register in November 2015 with registration number CRD42015029536.

## Background

Intravenous regional anesthesia (IVRA) or Bier Block is a simple and effective but underused anesthetic technique for hand and forearm surgery [1–5]. This technique, introduced by Dr. August Bier in 1908, provides complete anesthesia as well as a bloodless field during surgery [6]. Traditionally, an upper arm tourniquet has been used to sequester the local anesthetic and to create a bloodless surgical field [7]. Major complications after IVRA with an upper arm tourniquet are rare but are mostly related to local anesthetic systemic toxicity after release of the tourniquet [8]. Symptoms of a major systemic local anesthetic reactions include convulsions, coma, respiratory depression and arrest and cardiovascular depression with possible fatal consequences. Therefore, some clinicians prefer other locoregional techniques or even general anesthesia for hand and forearm surgery.

Use of a forearm tourniquet has been introduced in 1978 by Rousso et al. [9, 10] and comes with the big advantage of lower (non-toxic) local anesthetic dosage requirement to produce a good quality of analgesia [6, 11, 12]. Consequently, there is no minimal tourniquet inflation time after forearm IVRA. In addition, it has been postulated that sensory onset time after forearm IVRA may be shorter than after upper arm IVRA [13–15]. With these two features, forearm IVRA may be the ideal anesthetic technique for short ambulatory surgery of hand and wrist. Finally, it has also been suggested that a forearm tourniquet elicits less ischemic pain and therefore can be tolerated longer with less need for additional analgesia or sedation and lesser chance for the need of conversion to general anesthesia [16]. Despite these advantages, forearm IVRA is still not widely applied because it was thought that the interosseous vessels in the forearm might not be occluded during the procedure with a potential risk of incomplete hemostasis and leakage of local anesthetic into the circulation [11, 17]. Nevertheless, several studies have refuted that idea and have revealed that forearm IVRA is safe and effective [6, 18, 19].

In clinical practice, the optimal anesthesia technique for surgery of the distal extremity is still undecided. Recently, some studies compared the analgesic efficacy and side-effects of IVRA with a forearm tourniquet to the conventional upper arm tourniquet, in adults undergoing surgery of the distal extremity. The aim of our paper was to perform a systematic review and meta-analysis of these randomized controlled trials to synthetize the best evidence for this topic.

## Methods

The methods used in this review, including literature search strategies, study selection criteria and data extraction and synthesis, are outlined subsequently. This systematic review was performed in accordance with the Preferred Reporting Items for Systematic Reviews and Meta-Analyses (PRISMA) guidelines. A review protocol was published in the PROSPERO register (http://www.crd.york.ac.uk/PROSPERO) in November 2015 with registration number CRD42015029536.

### Study identification

The PubMed Central, MEDLINE, EMBASE and Cochrane Central Register of Controlled Trials (CENTRAL) databases were searched for relevant articles between December 2015 and April 2016. There was no restriction on publication date. The search was limited to articles written in English, French, Dutch, German and Spanish and was complemented by hand check of reference lists of reviews and included RCT’s for additional relevant studies. Initially, the following keywords were used in combination with the Boolean operators AND or OR: [“intravenous regional anesthesia” OR “bier block” AND (modified OR forearm cuff OR forearm tourniquet)]. These keywords were too specific. Therefore, the search was broadened by using the search term ‘Intravenous regional anesthesia’ without any restrictions.

### Eligibility criteria

Studies meeting the following criteria were considered eligible for inclusion: (1) Published RCT’s and quasi-controlled trials; (2) Adult patients undergoing procedures on the upper extremity. Experiment group received modified intravenous regional anesthesia with a single forearm cuff and control group received conventional Bier block with an upper arm cuff. Studies were excluded if less than five patients per group were involved. Case reports, reviews and conference abstracts were also excluded.

Finally, all prospective and retrospective RCT’s and cohort studies, studying IVRA with a forearm cuff were included in a parallel analysis in an attempt to study the incidence of complications (i.e. signs of local anesthetic systemic toxicity or other complications) after forearm IVRA.

### Data collection and data extraction

Two authors (VD and YH) initially screened article titles independently. Abstracts of potentially relevant articles were subsequently assessed, and those without relevance were eliminated. Full-text manuscripts of all remaining studies were obtained, read and assessed qualitatively. Study quality was evaluated by using the Cochrane risk of bias tool for assessing risk of bias. Interrater variability and discrepancies were resolved by discussion with a third party (JPO). The search was complemented by hand check of reference lists of reviews and included RCT’s for additional relevant studies. The risk of bias was assessed using the Cochrane risk of bias tool. Where necessary, authors were contacted to obtain further detailed information.

We set out to retrieve data according to the following primary outcome measure: (i) success rate of IVRA. We defined block success rate as the percentage of blocks which allowed patients to undergo surgery without conversion to general anesthesia (i.e. good or excellent anesthesia). The primary hypothesis was that forearm IVRA is equally effective in providing a surgical block as upper arm IVRA. Secondary outcome measures included: (ii) onset time of sensory block; (iii) Tourniquet tolerance time and incidence of tourniquet pain necessitating additional sedative; (iv) complications associated with forearm IVRA; (v) Choice of local anesthetic and dosage of local anesthetic were noted. The Visual Analogue Scale (VAS) is used to describe the level of discomfort or pain caused by the tourniquet.

### Statistical analysis

Information about study design, participants, intervention with choice and dosage of local anesthetic, surgical procedure, tourniquet placement and incidence of adverse outcomes associated with IVRA was tabulated. We reported the pooled risk ratio with 95% confidence interval for the binary outcome “block success rate”. Synthesis was done using RevMan (Review Manager 5.2). Statistical heterogeneity was calculated using Chi Square and also the 퐼^2^-test to describe the percentage variability in individual effect estimates that could be due to true differences between the studies rather than a sampling error. Study findings were also documented in the form of a “Summary of Findings” table. We did not perform subgroup analyses since the patient population in the selected studies was too small.

## Results

**Fig. 1:**
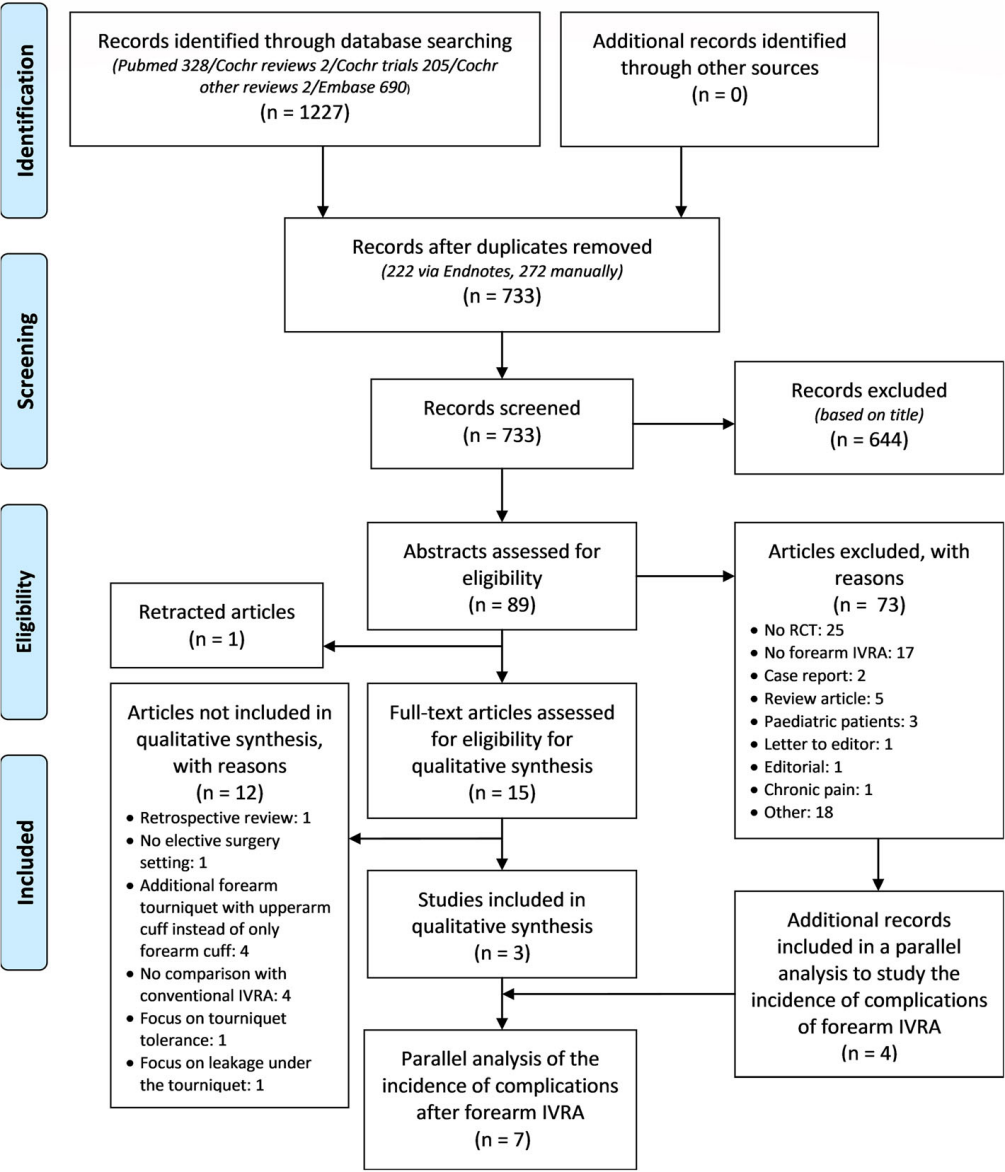
PRISMA flow diagram. Abbreviations: Cochr = Cochrane Library, RCT = randomized clinical trial, LA = local anesthetic, IVRA = intravenous regional anesthesia

A PRISMA flow chart with the search results is presented in Fig. 1. Out of 1227 items, 733 records were obtained after removing duplicates. Based on title and abstract, 717 records were excluded. One article was retracted because of fabricated data and 15 full-text articles were assessed. After exclusion of ineligible studies, 3 RCT’s were included in the qualitative and quantitative synthesis [7, 20, 21]. Finally, 4 articles were added into a parallel analysis of 7 reports that provided data on the incidence of complications and success rate after forearm IVRA.

**Fig. 2:**
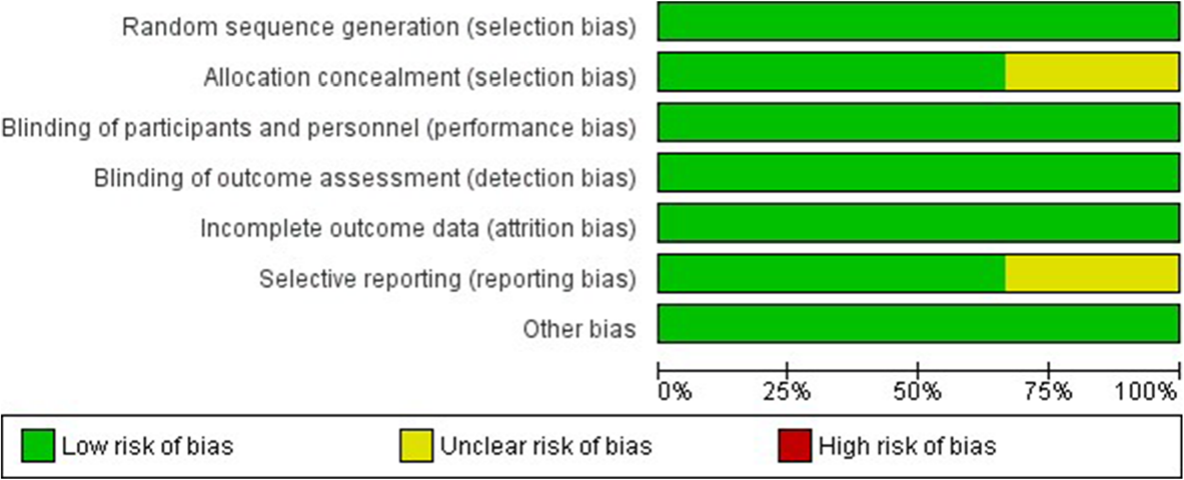
Risk of bias across studies assessed using the Cochrane risk of bias tool

**Fig. 3:**
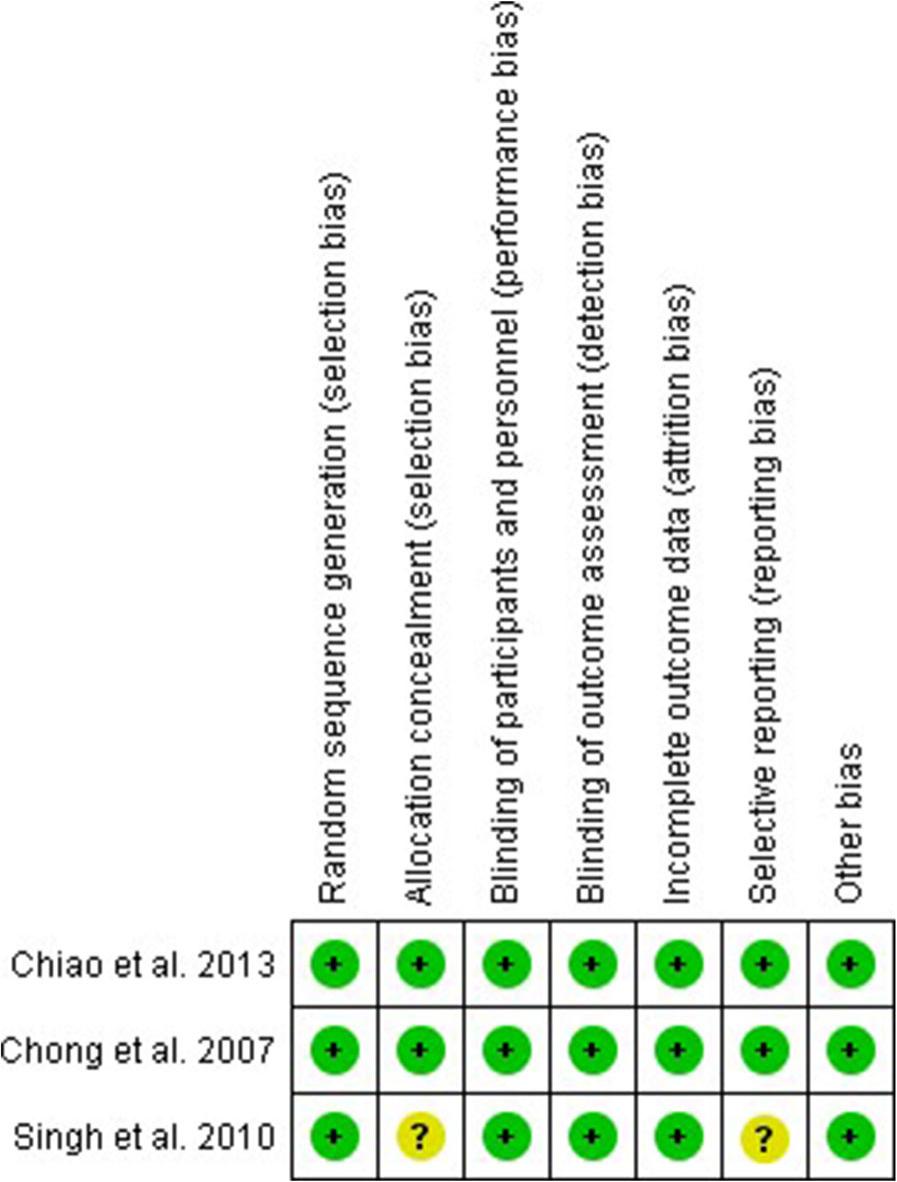
Risk of bias in individual studies assessed using the Cochrane risk of bias tool

The risk of bias across studies is shown in the bar graph (Fig. 2) obtained through Review Manager 5.3. The risk of bias in individual studies is presented in Fig. 3. Two studies [7, 20, 21] had a low risk of bias in all domains. Considering the nature of the interventions, blinding of participants was not possible. However, outcome-assessors were blinded to treatment allocation (observer-blinded study) in all 3 studies.

Characteristics of study design, participants, intervention with choice and dosage of local anesthetic, surgical procedure and tourniquet placement are listed in Table 1. The oldest study [21] administered the same doses of LA for both the upper arm and the forearm group. The other two studies [7, 20] administered half the dose of LA for forearm IVRA in comparison with upper arm IVRA.

**Table 1:** Summary of Findings table of included studies

	Chong et al., 2007	Singh et al., 2010	Chiao et al., 2013
Study design	RCT, 2 groups, parallel design JADAD score: 4/5	RCT, 2 groups, parallel design JADAD-score: 5/5	RCT, 2 groups, parallel design JADAD-score: 5/5
Participants	30 patients with a distal radius fracture which required manipulation and reduction. • Standard Bier’s block: Mean age: 56.9 ± 20.1 M/F: 7/8 • Modified Bier’s block: Mean age: 48.5 ± 20.6 M/F: 9/6	40 ASA I-II patients who were undergoing hand or forearm surgery. • Standard Bier’s block: Mean age: 29.8 ± 8.1 M/F: 13/7 • Modified Bier’s block: Mean age: 36.0 ± 11.0 M/F: 11/9	59 ASA I-III patients having distal upper extremity surgery under IVRA. • Standard Bier’s block: Mean age: 40.1 (22–66) M/F: 7/21 • Modified Bier’s block: Mean age: 40.8 (22–67) M/F: 10/18
Interventions	IVRA with upper arm cuff against forearm cuff, same dose of LA. IVRA was performed with: • 3 mg/kg of 1% lidocaine made up to 40 mL of solution in both study groups.	IVRA with upper arm cuff against forearm cuff, upper arm gets double dose LA compared to forearm. IVRA was performed with: • 3 mg/kg of 0.5% lidocaine with 0.3 mg/kg ketorolac in the upper arm group. • 1.5 mg/kg of 0.5% lidocaine with 0.15 mg/kg ketorolac in the forearm group.	IVRA with upper arm cuff against forearm cuff, upper arm gets double dose LA compared to forearm. Sedation administered if VAS > 4. IVRA was performed with: • 15 ml of 2% lidocaine and 20 mg ketorolac in the upper arm group. • 8 ml of 2% lidocaine and 10 mg ketorolac in the forearm group.
Surgical procedures	Manipulation and reduction of closed distal radius fractures.	Ganglion excision (3/2), contracture release (2/4), excision biopsy (3/3), open reduction and internal fixation of single bone forearm fracture (5/7), closed reduction and internal fixation (2/0), carpal tunnel release (1/1), foreign body removal (1/1), external fixator application (2/2), nerve repair (1/0) * (upper arm group/forearm group)	Surgeries in each group were similar and were completed without complications. Surgeries included ganglion cyst excision, mass excision, digital nerve repair, metacarpal and digital fracture pinning, and ORIF, ruptured tendon repair, and palmar fasciotomy.
Outcomes	Pre-block and manipulation VAS scores, outcome of the reduction, complications, incidence of LA toxicity.	Quality of surgical anesthesia, sensory onset and regression of block, proximal tourniquet tolerance, VAS at 30 and 60 min. Postoperatively, postoperative analgesic use, incidence of LA toxicity, local complications (tourniquet).	Sedation need based on intraoperative VAS score, PACU bypass.
Tourniquet placement	An upper arm and forearm cuff was applied in all cases in an attempt to blind the patient. Only one of both cuffs was inflated. IVRA with upper arm cuff against forearm cuff, same dose LA. • Standard Bier’s block: Inflation of the upper arm cuff. • Modified Bier’s block: Inflation of the forearm cuff.	• Standard Bier’s block: A pneumatic double-cuffed tourniquet (14-cm wide) was placed on the upper operative arm at the point of maximum diameter. • Modified Bier’s block: The same tourniquet was positioned 5 cm below the medial epicondyle on the forearm.	The single-cuff pneumatic pressure tourniquet was placed immediately above or below the elbow crease and on the top of a circumferentially placed cotton cast padding before inflation.
Notes	1 patient in the forearm group was admitted for open reduction and internal fixation after failed reduction.		3 patients did not receive allocated intervention: • *n* = 2: given narcotic during exsanguinations of arm • *n* = 1: surgeon released tourniquet right after block placement

*RCT* randomized clinical trial, *ASA* American Association of Anesthesiologists physical status classification system, *M/F* male/female ratio, *IVRA* intravenous regional anesthesia, *LA* local anesthetic, *VAS* visual analog scale, *PACU* post anesthesia care unit

### Block success rate

**Fig. 4:**
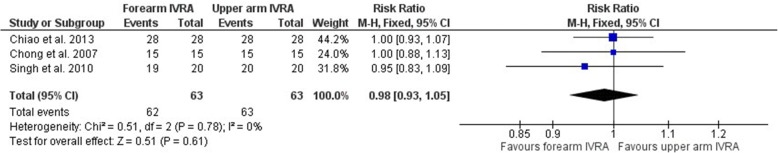
Forest plot for block success rate. Abbreviations: IVRA = intravenous regional anesthesia, CI = confidence interval

All three studies [7, 20, 21] investigated the analgesic efficacy of forearm IVRA compared to conventional upper arm IVRA. Singh et al. [20] described one patient in the forearm group where conversion to general anesthesia was needed because of lack of adequate sensory block. Good or excellent anesthesia was achieved in all other patients. After pooling of the results (*I*^*2*^ = 0%; no heterogeneity), we could not find a difference in efficacy between the two techniques with a calculated RR of 0.98 [0.93, 1.05] (Fig. 4).

All studies reporting on the analgesic efficacy of forearm IVRA are listed in Table 2. Forearm IVRA was associated with a very high success rate of 99.5% in a cohort of 383 patients (Table 2).

**Table 2:** Incidence of complications and block success rate in patients receiving a forearm IVRA

	Type of study	Patients with signs of local anesthetic systemic toxicity/total number of patients receiving forearm IVRA (%)	Local anesthetic + dosage	Success rate of forearm IVRA (%)
Studies using a full dose of la (type of study)				
Chong et al., 2007	Prospective RCT	1/15 (6.7%)	Lidocaine 1% - 3 mg/kg made up to 40 ml of solution	15/15 (100%)
Studies using a lower dose of la (type of study)				
Chan et al., 1987	Prospective study No control group	0/55 (0%)	Lidocaine 0.5% - 2 mg/kg with a maximum volume of 20 ml	55/55 (100%)
Peng et al., 2002	Prospective RCT	0/40 (0%)	Lidocaine 0.5% or Ropivacaine 0.375% - 0.4 ml/kg with a maximum volume of 25 ml	40/40 (100%)
Karalezli et al., 2004	Prospective study No control group	0/120 (0%)	Prilocaine – 1.5 mg/kg in 10 ml	119/120 (99.1%)
Arslanian et al., 2013	Retrospective study	0/105 (0%)	Lidocaine 0.5% - 25 ml	105/105 (100%)
Singh et al., 2010	Prospective RCT	0/20 (0%)	Lidocaine 0.5% - 1.5 mg/kg	19/20 (95%)
Chiao et al., 2013	Prospective RCT	0/28 (0%)	Lidocaine 2% - 8 ml (+ 10 mg ketorolac)	28/28 (100%)
Total low dose forearm IVRA		0/368 (0%)		366/368 (99.4%)
Total all IVRA		1/383 (0.26%)		381/383 (99.5%)

*IVRA* intravenous regional anesthesia, *RCT* randomized clinical trial, *LA* local anesthetic

### Onset time of sensory block

Only Singh et al. [20] investigated the onset time of sensory block. Despite a tendency towards faster onset in the forearm IVRA group, Singh did not find a difference between the two groups.

### Tourniquet tolerance time and incidence of tourniquet pain necessitating additional sedatives

Tourniquet tolerance was described in 2 of 3 articles. Singh et al. [20] investigated tourniquet tolerance time which was defined as the time required for the tourniquet pressure to become painful (VAS > 3). Singh et al. concluded that tourniquet tolerance time was longer with a forearm tourniquet. Chiao et al. [7] concluded that tourniquet tolerance time was much longer after forearm IVRA. More specifically, mean VAS score rose to 3 after 10 min and above 4 after 40 min in the upper arm group versus a mean VAS of 0.5 after 10 min and less than 1.5 after 40 min. Deep sedation with propofol (started when patients intraoperatively reported a VAS score > 6) was necessary in 22 patients after upper arm IVRA versus in 1 patient after forearm IVRA.

### Complications associated with forearm IVRA

All studies reporting on the incidence of complications associated with forearm IVRA are listed in Table 2 [6, 7, 11–15, 20–24]. From a total of 383 patients receiving forearm IVRA, only 1 patient (0.26%) reported signs of local anesthetic systemic toxicity (perioral numbness) [21]. No other complications were noted.

## Discussion

In the present systematic review, a forearm IVRA was found to be equi-effective in providing a surgical block as compared to a conventional upper arm IVRA, even if a reduced dose of local anesthetic was administered. Furthermore, the onset of sensory block tended to be faster and patients experienced less tourniquet pain after forearm IVRA. Finally, forearm IVRA was found to be a very safe procedure as no severe occurred in a cohort of 383 patients receiving forearm IVRA.

Forearm IVRA is still not widely applied because of a potential risk of incomplete hemostasis and leakage of local anesthetic into the circulation through the interosseous vessels [11, 17]. The studies included in our systematic review did not report on the occurrence of incomplete hemostasis. However, mild signs of local anesthetic systemic toxicity due to leakage of local anesthetic into the systemic circulation was noted in 1 of 383 patients (0.26%). However, in the subgroup of 368 patients receiving a reduced dose of local anesthetic, no case of systemic local anesthetic toxicity was noted. Furthermore, Coleman et al. [18] already investigated the leakage of a radiolabeled substance with a structure similar to lidocaine in a crossover study comparing a forearm with an upper arm tourniquet. The leakage of radiolabeled substance during inflation was found to be similar in both groups. After deflation, mean loss of radioactivity was higher in the upper arm tourniquet group (*P* < 0.001) because this group received a higher dose. They concluded that forearm IVRA results in tourniquet leakage comparable to conventional IVRA and is potentially safer because the required dose of local anesthetic is smaller.

Another postulated contraindication for the use of a forearm tourniquet is the risk of peripheral nerve damage [25]. Sanders stated that “the tourniquet is most safely applied to that part of the limb which is of maximum circumference, and well-padded with periosseus muscle”. However, our results indicate that these presumptions are incorrect as no single peripheral nerve injury is described in the studies included in this systematic review. We do however realize that peripheral nerve injury is very rare and the patient population of this systematic review is probably too small to reveal this complication.

Conventional IVRA has become less popular because of the risk of (accidental) loosening of the tourniquet with potentially life threatening systemic toxicity of the local anesthetic [8]. This risk however can be avoided by the use of smaller non-toxic doses of local anesthetic in forearm IVRA.

In literature, it is suggested that other benefits of forearm IVRA as compared to a traditional upper arm IVRA include a better tourniquet tolerance and a drier surgical field. Our results on tourniquet tolerance echo those of a trial comparing upper and forearm tourniquet tolerance time in healthy volunteers [26]. In this trial, healthy volunteers also tolerated a forearm cuff longer than an upper arm cuff. In another RCT, Frank et al. [23] showed that a forearm tourniquet is associated with a drier surgical field with less oozing as compared to an upper arm tourniquet.

Use of an additional forearm tourniquet together with a conventional upper arm IVRA has also been described in literature [14, 15, 22, 23]. These RCT’s administered the same dose of local anesthetic in both the conventional upper arm IVRA group and the additional forearm tourniquet. These studies all demonstrated that use of an additional forearm tourniquet is associated with a more rapid onset of sensory block, a similar or even better quality of anesthesia as well as a lower incidence of local anesthetic toxicity as compared to the conventional technique.

The optimal dose and type of local anesthetic for this modified forearm block is still undecided. Peng et al. [24] randomized 51 patients undergoing outpatient hand surgery to receive forearm IVRA with either 0.4 ml/kg of ropivacaine 0.375% or 0.4 ml/kg of lidocaine 0.5%. Onset time of anesthesia and motor block were found to be similar in both groups.

There are several limitations to this systematic review. First, we could only identify a small number of trials with relatively few patients meeting the inclusion criteria. None of these studies however showed a high risk of bias. Furthermore, all of these studies concluded that forearm IVRA is as effective as upper arm IVRA in providing adequate anesthesia. Second, heterogeneity is a real concern given the use of different types and doses of local anesthetic. Future research should focus on the identification of the optimal type and dosage of local anesthetic to perform a forearm IVRA. The ideal dosage should provide fast onset of an effective surgical block without exceeding the toxic level.

## Conclusion

In conclusion, this systematic review demonstrates that forearm IVRA is as effective in providing a surgical block as compared to a conventional upper arm IVRA, even with a reduced, non-toxic dosage of local anesthetic. No severe complications were associated with the use of a forearm IVRA. Therefore, the main advantage of a forearm IVRA with a reduced dose of local anesthetic is the high safety profile compared to conventional upper arm IVRA. Other benefits of the modified technique may include a faster onset of sensory block, better tourniquet tolerance and a dryer surgical field. Future studies should focus on finding the optimal dosage of local anesthetic, investigate the economic benefit of a potential PACU bypass (e.g. no sedation/general anesthesia needed; low doses local anesthetic), and could compare this technique with other locoregional anesthesia techniques (e.g. forearm block) for forearm and hand surgery.

## Abbreviations

ASA: American association of anesthesiologists physical status classification system
Cochr: Cochrane Library
IVRA: Intravenous regional anesthesia
LA: Local anesthetic
M/F: Male/female ratio
PACU: Post anesthesia care unit
PRISMA: Preferred reporting items for systematic reviews and meta-analyses
RCT: Randomized clinical trial
RevMan: Review Manager
VAS: Visual analog scale
